# Broadening Our Understanding of Farm Children’s Risk Exposure by Considering Their Parents’ Farming Background

**DOI:** 10.3390/ijerph18105218

**Published:** 2021-05-14

**Authors:** Florence Becot, Casper Bendixsen, Kathrine Barnes, Josie Rudolphi

**Affiliations:** 1 National Farm Medicine Center, Marshfield Clinic Research Institute, 1000 N Oak Ave, Marshfield, WI 54449, USA; bendixsen.casper@marshfieldresearch.org (C.B.); barnes.kate@marshfieldresearch.org (K.B.); 2 Agricultural and Biological Engineering, University of Illinois at Urbana-Champaign, Urbana, IL 61801, USA; josier@illinois.edu

**Keywords:** farm children, risk exposure, farming background, farm safety beliefs and adoption of practices, parenting, social and economic factors, socialization and social norms

## Abstract

While farm safety researchers have seldom considered the association between farm parents’ background and their children’s safety, researchers who have compared first- and multi-generation farmers have found differences that may shape safety outcomes. We draw on the farm safety and family farm bodies of literature and a survey of 203 United States farm parents to assess the role of farming background in farm children risk exposure. Exploratory in nature, the bivariate analysis revealed no statistically significant differences between first- and multi-generation farmers in children injury, agricultural safety perceptions, knowledge, and practices but revealed differences in key demographic characteristics and parenting styles. A range of factors likely explain these findings with meso- and macro-level factors likely impacting farm parents’ ability to adopt safety practices. In contrast to the emphasis on knowledge and behaviors, we call for the integration of lived realities in farm safety research and to do so in a way that connects realities and choices to larger contexts. We also call on the need to expand the toolkit of interventions to address meso- and macro-level factors. A shift towards addressing social and economic conditions in agriculture could reduce farm children’s injuries while supporting the sustainability of farm labor systems.

## 1. Introduction

In the United States of America (USA), farm children are exposed to significantly more risks of injury and fatality than the general population [1,2,3,4]. Every day, about 33 children are seriously injured in agricultural-related incidents, and about every three days, a child dies [1,5]. Furthermore, about 60% of agricultural-related injuries are sustained by non-working, by-standing children who live on farms [1]. Farm-related fatalities constitute almost half of all fatalities of all working youth [6]. The high-risk exposure of farm children compared to children in the general population has been explained by several key differences: the overlap of farm children’s home with their parents’ worksite and federal labor laws that permit children to work on their parents’ farm at any age and that permit children younger than 10 to work on non-family-owned farms if the farm is exempt from paying federal minimum wage [7,8,9,10,11,12]. Besides direct health and safety impacts, ensuring the safety of farm children is directly connected to the social and economic sustainability of farm labor systems. Supervising children while doing farm work can reduce farm parents’ productivity [13,14]. Juggling multiple roles can also be stressful [13,15], and stress is associated with higher rates of injury [16,17]. Furthermore, in early stages of the farm business, which have traditionally overlapped with the birth and early years of their children, these farm families are more likely to be in vulnerable positions due to high financial demands [15,18,19,20,21]. These high financial demands may limit farm parents’ abilities to adopt recommended farm safety practices such as the use of physical barriers on the farm or child supervision off the worksite through the use of childcare [22,23].

For over three decades, farm safety scholars have sought to identify the factors associated with variations in farm children’s risk exposure to develop interventions to address these risks. They have assessed the role played by demographic characteristics of farm parents’ (gender, number of children, off-farm employment, and educational attainment) [24,25,26,27,28,29,30,31,32] attitudes, norms, and parenting styles [24,26,28,29,33,34,35,36,37,38,39], characteristics of farm operation (namely, the type of commodity produced, scale of operation, and more recently, marketing channels and growing practices) [25,39,40,41]. An extensive body of work has also considered the intersection between farm risk exposure and farm safety practices with children’s demographics (specifically age and gender) [24,25,30,32,40,41,42,43,44,45], safety equipment used, tasks given, and presence on the farm [25,26,31,37,40,43,46,47,48,49,50]. While this literature has led to the development of an in-depth understanding of which farm children, doing what tasks, and supervised by whom are most at risk, much of the farm safety research has been conducted either implicitly or explicitly, from the vantage point of farmers who grew up on a farm (i.e., multi-generation farmers). The extent to which farm safety beliefs, knowledge, and practices are shaped by farm backgrounds has, to our knowledge, not been explicitly assessed.

In the broader family farm literature, which focuses on farm operations that are owned and/or operated by a family, whereas the home and workspace still tend to overlap, scholars have begun to assess differences between first- and multi-generation farmers and the consequences of these differences on the sustainability of family farms. This work has largely been driven by the structural barriers to entry into agriculture along with the development of programs and policies to encourage new entrants into agriculture. Comparing farm income diversification [51], enterprise growth, adaptation, and reproduction [52], land transition and knowledge acquisition [53], climate change adaptation [54], social networks [55], and childcare access [13,14], family farm scholars have found differences between these two groups of farmers in socialization into agriculture, access to resources, and farming knowledge and skills. In turn, these differences shape farm goals, farm structures, and the adoption of farm production practices [51,52,53,56]. Thus far, this body of work reveals a greater level of vulnerability among first-generation farmers due to lower access to financial and social resources and steep learning curves [53,54,55]. This work also highlights the role played by broader social, economic, and policy factors in shaping access to resources [13,14]. With knowledge and access to resources shaping exposure to risk on the agricultural worksite [24,57], these differences may also affect a range of outcomes related to the health and safety of everyone on the farm, which would then call for updated farm safety interventions. The need to refine and develop farm safety interventions to both target a variety of leverage points within the complex agri-family system and account for the needs and realities of the heterogeneous farm population is essential [9,58]. Historically, farm safety interventions tend to over-emphasize individual-level interventions (i.e., knowledge-deficit and behavioral change models) despite evidence of their ineffectiveness, since they do not address root challenges and can be counterproductive [9,23,59,60,61].

In this article, we draw on insights from the merging of farm safety and the broader family farm literature along with primary survey data from 203 farm parents in two US states—Pennsylvania (PA) and Wisconsin (WI)—to assess differences between first- and multi-generation farmers on a range of variables connected to farm children’s risk exposure. Due to the limited consideration of the role of farming background in the farm safety literature and our small sample size, our study is exploratory in nature. Consistent with the family farm literature, we define first-generation farmers as those that did not grow up or inherit a farm operation; we define a multi-generation farm as one where more than one generation of a family has been an owner–operator [52,53,54]. Before we present our empirical case, we summarize key differences between first- and multi-generation farmers identified in the family farm literature. As we summarize differences from this body of work, we also incorporate insights from the farm safety literature since these two bodies of work drive our analysis and discussion of the findings.

## 2. Literature Review

### 2.1. Differences in Socialization

The socialization process into agriculture has implications for children’s involvement in farm work, farmers’ commitment to the farm operation, and farm goals. Family farm scholars have discussed the ways socialization into agriculture of first- vs. multi-generation farms differs. Socialization on multi-generational farms often happens by bringing children to the worksite so they cultivate an appreciation for the work, develop their skillset, and contribute to the farm enterprise [13,14,52]. Meanwhile, first-generation farm parents are less likely to require their children to work on the farm. Instead, these parents emphasize that their children should choose their own career path [13,62]. Still, they value the farm as a positive environment to raise their children [13,62]. These findings align with the farm safety literature, which provides insights into the intersection between the socialization process in agriculture and exposure to risk. While farm parents often recognize the dangers associated with farm work, farm safety scholars have found that social and cultural pressures connected to farm traditions and farm succession are associated with engaging children in farm work and increased exposure to risk [24,28,31,33,36]. Furthermore, the socialization process can normalize danger with implicit agreement surrounding acceptable farm danger and risk [9], which then extends into adulthood. The long-term commitment developed by multi-generation farmers is associated with a strong drive to carry on the farm legacy, leading these farmers to go to great lengths, including redirecting resources from the household to the farm business, shouldering heavy workloads and risk-taking [9,19,52,63]. While multi-generation farmers might have internalized the importance of farm safety practices through witnessing or being a victim of a farm incident, findings on prior near misses altering safety practices in the long-term are mixed [9,28]. Free from historical farm legacies, first-generation farmers tend to have more freedom in how they structure their farm operation and do not feel as much pressure to pass the farm on to the next generation [52], which may lead to less risk-taking. However, because they were raised in an environment that likely did not carry the same level or type of risks, these first-generation farmers may not be as familiar with risk, and there may be a mismatch between the parenting style they internalized in non-farm places and the realities of on-farm child rearing. Furthermore, and potentially similar to findings with farm women [9], first-generation farmers wanting to prove themselves as “authentic farmers” might adopt unsafe practices.

### 2.2. Acquisition of Technical Farming Skills and Adoption of Practices

The family farm literature points to nuanced and contradictory findings across first- and multi-generation farmers around the acquisition of technical farming skills once they assume management duties and the extent to which farmers are willing and able to adopt new practices. First-generation farmers often face a steep learning curve to acquiring and mastering farming skills [53,56,64]. This lack of technical knowledge places first-generation farmers at a disadvantage for productivity and economic viability of their farm [56]. Compared to multi-generation farmers, first-generation farmers tend to rely on mentors or internet searches and are less likely to join industry groups and use state extension services [54,65]. Looking at drought adaptation strategies, Munden-Dixon, Tate, Cutts and Roche [54] found that lower levels of expertise and experience led first-generation farmers to take more risks in their approach to production. Due to their extensive farming knowledge, multi-generation farmers might not be willing or able to change practices they deem adequate for their operation. This is due to several factors including social pressures of continuing tradition, path dependency created by farm investments, and debt that renders changes to farm structure and marketing channels difficult and/or expensive [53,66]. This could also be due to multi-generation farmers using experience as a justification for both risk-taking and safety efforts. Lastly, without making a distinction on farm background, the farm safety literature also found evidence of practices being passed down through generations [24,25,33], farmers’ awareness of the nature and sources of dangers [23], and farm parents reporting that they know best about their children’s maturity and safety [9,28,50].

### 2.3. Access to Resources

The farm safety literature has established connections between access to resources—mainly financial—and farm safety. In particular, financial difficulties are associated with both children working more on the farm [24,25,28,40,46] and a decreased ability to adopt farm safety recommendations including the use of childcare [8,28,31,40,57]. Likely to shape the uptake of farm safety practices, the family farm literature has found that families in the early stages of starting or taking over a farm tend to overlap with key reproductive stages. In these early stages, young farm parents must contend with competition for financial resources and time between the development needs of the household and the operation [14,19,20,67,68,69]. Furthermore, high demands on younger and beginning farmers are associated with higher levels of financial and mental stress [18,21]. This body of work has found some variations in access to resources between first- and multi-generation farmers. First-generation farmers faced difficulties associated with needing significant financial resources to establish their farm operation [13,51,53]. Social networks have also been found to play an essential role in mediating access to resources. For first-generation farmers, a lack of social network and trust in their new communities impedes access to land, credit, markets, and labor [13,53,55]. The lack of financial and social resources also impacts childcare arrangements. First-generation farmers reported greater difficulties with accessing and paying for childcare compared to multi-generation farmers. This has in part been explained by first-generation farmers having moved away from their families to access farmland, which decreased their ability to rely on family and community networks to provide childcare [13,14].

### 2.4. Summary

The synthesis of family farm literature on first- and multi-generation farmers and farm safety literature hints at numerous, and potentially contradictory, implications on the safety of farm children across these two groups. First-generation farmers, in contrast to multi-generation farmers, appear to have lower access to financial and social resources, while they may be more receptive to changing farm practices in line with safety recommendations. These factors may lead to different levels of risk exposures. In this article, we begin to tease out some of the differences between the two groups as they pertain to farm parents’ safety practices exposing farm children to risk.

## 3. Materials and Methods

### 3.1. Research Design

Our data are from a survey of farm parents in PA and WI aimed at understanding their attitudes, parenting styles, and practices towards the safety of their children. This research presents original bivariate analysis to assess the differences between first- and multi-generation farmers and is a companion study with Rudolphi, Barnes, Kieke, Koshalek and Bendixsen [35], which focuses on the association between parenting styles and farm children injuries. The survey instrument was developed collaboratively by an interdisciplinary team of researchers and included questions related to basic personal and household demographics, farm operation characteristics, the relationship with children, participation of children in farm activities, farm safety practices and knowledge, parenting style, and history of farm children’s injury. Parenting style questions were based on a widely used instrument [70], while confidence in agricultural safety and health questions were based of previous research and a child/youth agricultural safety checklist [22,71]. The survey instrument is available upon request. The study was conducted in accordance with the Declaration of Helsinki. The research protocol BEN20314 was exempt from review by the Marshfield Clinic Research Institute Institutional Review Board on August 21, 2014. Research participants provided their informed consent to participate in the research study by mailing back the survey.

### 3.2. Recruitment and Data Collection

We collected data using a modified Dillman approach for mail surveys [72]. The survey sample frame was PA and WI farmers over the age of 18 and reporting farming as a full- or part-time occupation. PA and WI were selected as study states due to the continued prevalence of long-established family-owned and -operated farms. We drew a proportional random sample of 998 farmers from four lists: the PA’s Department of Agriculture licensed dairy producers and private pesticide applicators and the WI’s Department of Agriculture, Trade and Consumer Protection licensed dairy producers and private pesticide applicators. The dairy producers list was used due to the high proportion of dairy farmers in these two states, whereas dairy farms are conducive to the continued overlap of the home and work sphere due to the need to be nearby for milking. This is different than the growing number of new, very large company-owned dairies in Texas, Idaho, and California that rely on employees. The pesticides applicator list was used to reach farmers who produce a range of crops. Non-agricultural producers were removed from the pesticide applicator list before the random sample was drawn. Between February and April 2018, we sent the initial mailing including a cover letter, the survey instrument, a postage paid return envelope, and a $5 bill. Approximately two to three weeks later, we followed up with a postcard reminder, and two to three weeks later, we sent another mailing of the survey instrument with a postage paid envelope. We received 470 completed surveys, leading to a 47% response rate. In this paper, our analytical sample is limited to the 203 respondents who reported having at least one child.

### 3.3. Survey Measures and Recoding

The full list of survey measures, measurements, and recoding is included in Table 1. We established first- vs. multi-generation farmer status using a question that asked respondents which generation of the farm operation they were from. To ensure at least five observations per cell in the bivariate analysis, we recoded five variables: educational attainment (from four to three categories), off-farm work (from three to two categories), physical and invisible boundaries (from five to two categories), and perception of safety in agriculture (from three to two categories). Respondents were asked about the age, gender, and involvement in farm work for each child. To simplify the analysis, we created two new variables by summing responses for all children: number of children (continuous variable) and children participate in farm work (dummy variable). Additionally, we created a new dummy variable for having children under seven. The beginning farmer variable was created using the number of years farming variable using 10 years of experience or less as the threshold for being categorized as a beginning farmer. In terms of commodities, the dairy or beef variable was creating using two separate variables, the other variable was created using four separate variables (swine, sheep, poultry, other).

The confidence in ability to supervise children and in farm safety knowledge variables was determined through 10 four-point scale items (1 = strongly disagree; 4 = strongly agree). We created new variables by summarizing these 10 items (see Table 2 for items). The Cronbach’s alpha scores to assess internal consistency of the items were above the commonly accepted threshold of 0.7 (0.92 for confidence in ability to supervise children and 0.84 for confidence in farm safety knowledge) [73,74]. To create the parenting style variable, we used the same approach as Darling and Steinberg [70] and Rudolphi, Barnes, Kieke, Koshalek and Bendixsen [35]. The parenting style measure is based on 15 five-point scale items (1 = strongly disagree; 5 = strongly agree) with two dimensions (parent involvement based on nine items and parent control based on six items) (see Table 2 for items). First, we summed up the items for the two dimensions; then, we calculated the mean. The sum for the parental involvement items ranged from 9 to 45 and the median value was 39. The sum for parent control items ranged from 6 to 30 and the median value was 27. Then, we created the new parenting style variable by using the median values as the threshold to determine what was high and low for each of the dimensions. The categories of the parenting style are as follows: authoritative (high on involvement and high on control), authoritarian (low on involvement and high on control), permissive (high on involvement and low on control), and uninvolved (low on involvement and low on control).

### 3.4. Analytical Strategy

To assess differences between first- and multi-generation farmers, we conducted bivariate analysis in STATA IC (version 15) (StataCorp LLC, College Station, TX, USA). We selected the bivariate over the multivariate analysis approach because regression models using children safety measures as dependent variables and the farming background variable as the independent variable controlling for demographic and farm characteristics from Table 1 failed basic model quality checks including not having at least five observations per cell in the crosstabs, the model *p* value was above the 0.05 threshold, or Hosmer–Lemeshow test indicated a poor model fit. While multivariate analysis is preferable, bivariate analysis is appropriate for exploratory studies such as ours.

We used Chi-square and ANOVA tests to assess statistically significant differences and the *p*-value threshold of ≤0.05 as the statistical significance threshold.

## 4. Results

### 4.1. Sample Characteristics

Among the 203 respondents, 32% were first-generation farmers; 68% were multi-generation farmers. Comparing demographic and farm characteristics, there were some differences across the two groups of farmers (Table 3). On average, multi-generation farmers were more likely to be male (81% compared to 69% of first-generation farmers, Chi^2^ = 3.7, *p* = 0.05); had a higher educational level (30% had at least a 2-year college degree compared to 15% of first-generation, Chi^2^ = 35.1, *p* = 0.00); had fewer children (average of 2.7 children compared to 3.7 children for first-generation, F = 9.7, *p* = 0.00); and had older children (47% had children under seven compared to 63% of first-generation, Chi^2^ = 4.2, *p* = 0.04). Multi-generation farmers also reported a different primary commodity focus with a higher production of field crops, although both groups had high rates of dairy or beef production (63% produced dairy or beef, 23% produced field crops, and 6% produced vegetables, fruits, or nursery plants, compared to 72% of first-generation farmers who produced dairy or beef, 11% who produced vegetables, fruits, or nursery plants, and 8% who produced field crops; Chi^2^ = 7.9, *p* = 0.05). Differences for the other demographic and farm characteristics between the two groups were non-significant. Respondents were on average 44 years old (F = 0.03; *p* = 0.86), 20% were beginning farmers (i.e., ≤ 10 years of farming experience) (Chi^2^ = 0.7; *p* = 0.40), 29% had an off-farm job (Chi^2^ = 3.2, *p* = 0.07), and they worked on average 60 h per week (Chi^2^ = 0.0; *p* = 0.96).

### 4.2. Farm Children Previous Experience with Injury

About one-third of all respondents reported that at least one of their children had suffered an injury at some point in their life that necessitated medical attention, with no statistically significant differences across the two groups (Chi^2^ = 0.6; *p* = 0.44) (Table 4). Male children were more likely to have been injured (68% of injuries were suffered by a boy) and 38% of injuries were suffered by children six years and younger (data not shown). All respondents but two provided information about the activity leading to the injury. Almost two-thirds (62%) of the activities leading to the injury were connected to farming equipment/environment, 24% to the playing or home environment, and 6% occurred off the farm/home site (i.e., school or daycare). Lastly, 8% of the explanations were too general to categorize the injury (i.e., was running, fell) (data not shown).

### 4.3. Farm Safety Beliefs, Knowledge, and Practices

Sixty-seven percent of all respondents believed agriculture is more safe or equally as safe as other occupations, with first-generation and multi-generation farmers appearing to share similar perceptions (Chi^2^ = 0.2; *p* = 0.67) (Table 4). Likewise, first-generation and multi-generation farmers reported a high level of confidence in their ability to supervise farm work (the overall score on a scale of 1 to 4 is 3.5 for the two groups; Chi^2^ = 2.96; *p* = 0.09) and a high level of confidence of safety knowledge (the overall score on a scale of 1 to 4 is 3.3 for the two groups; Chi^2^ =2.54; *p* = 0.11). Three-quarters of all farmers reported that their children participate in farm work (Chi^2^ = 0.2; *p* = 0.28). To keep children safe, 97% of farmers currently, or plan to, use an invisible play area (i.e., an area defined by a landmark on the farm or homestead that is reinforced orally) (Chi^2^ =0.1; *p* = 0.74), while the use of physical play areas was much lower, with 36% reporting a current or intended future use (Chi^2^ =0.4; *p* = 0.54).

### 4.4. Farm Safety and Parenting Styles

Authoritative style was the most common parenting style (42%), followed by uninvolved (34%), authoritarian (15%), and permissive (10%) (Chi^2^ = 12.5; *p* = 0.01). Parents with parenting styles characterized by high involvement (i.e., authoritarian and permissive), which is in turn associated with reduced risks of farm injury [35], was found in greater proportion among multi-generation farmers. Half of multi-generation farmers had an authoritative style compared to 24% of first-generation farmers, and 11% had a permissive parenting style compared to 8% of first-generation farmers.

## 5. Discussion

Our findings, based on 203 survey responses from farm parents in PA and WI, point to both similarities and differences between first- and multi-generation farm parent respondents connected to on-farm safety beliefs, knowledge, practices, and exposure to risk. We will now provide potential explanations to our findings, as well as the implications and avenues for future research, and we will do so by leveraging insights from both the farm safety and broader family farm literature. We will also discuss the limitations of our exploratory study.

### 5.1. Similarities between First- and Multi-Generation Respondents

We found no statistically significant differences between first- and multi-generation respondents in child injury, perceptions on agricultural safety and farm safety knowledge, and practices. Furthermore, we found a contradiction in our sample between farm safety perceptions and practices based on the previous literature that found an association between these factors and farm injury (see, for example, [29,36,37,38,75]). In particular, there was a high level of perceived safety in agriculture compared to other occupations and high level of confidence in farm safety knowledge and the ability to supervise. Yet, there was a seemingly high level of exposure to risk based on the proportion of children on the worksite through their involvement in farm work and low use of physical barriers—one of the most effective protective factors. Additionally, a third of surveyed farmers reported that at least one child had suffered an injury at some point in their life that required medical attention, and almost two-thirds of these injuries were directly connected to farming. While not directly comparable, estimates for all U.S. children indicate that 6.9% went to the emergency room for an unintentional non-fatal injury in 2019 [76].

The previous family farm literature on first- and multi-generation farmers hinted at numerous, and potentially contradictory, implications on the safety of farm children across the two groups. In particular, we had expected that first-generation respondents would have a lower level of confidence in their farm safety knowledge due to previous literature that has pointed to a knowledge differential between the two groups of farmers [53,54,64]. We had also expected that multi-generation respondents would be more likely to adopt safety practices, such as physical barriers, because they might be more attuned to the dangers on the farm. Yet, the high level of confidence and perception of safety are in line with previous findings that farm parents report knowing best about their children’s abilities and safety needs [9,28,50] and that farmers are aware of the nature and types of dangers [23,59,77]. Furthermore, previous studies have also found a similar contradiction between farm safety beliefs, knowledge, and practices [27,75,77].

The similarities between the two groups, along with the contradiction between beliefs and practices, point to three potential explanations. First, no matter the farming background, raising children is a deeply personal act connected to individual freedoms on how a parent wants to raise their children in sync with their values, realities, and knowledge of their children. Previous literature has found that raising children on the farm and involving them in the farm work is an important choice for both first- and multi-generation farmers [13,24,28,33,62]. Considering the focus of farm safety interventions on knowledge deficit and behavior change models [9,59,60,61], this potential explanation raises questions about whether farm parents are receptive to changing personal aspects of their lives. The findings also raise questions about the ways in which farm safety interventions could better incorporate farm parents’ motivations, expertise, and realities in program design. Scholars focusing on a variety of farm programming, including beginning farmer, women in agriculture, and farm safety, have made similar arguments [9,59,60,78,79,80]. In particular, they pointed to the problematic notion of knowledge hierarchies and have argued for the need to flatten these hierarchies between “experts” and farmers, because farmers are also experts in their own rights. These scholars have also argued for the need to ensure that farm programing is attuned and responsive to the values and realities of farmers. 

The second explanation, albeit very closely related to the first, is connected to knowledge biases. The farm safety literature has pointed to the overconfidence of farmers and their risk-taking with explanations connected to masculine identity, the prioritization of economic activity, and social norms around heavy workloads [9,24,31,33,77,81]. Murphy [82] has also discussed the farm safety-risk paradox, wherein farmers know that the work is dangerous, but they are still willing to take risks, and in some cases, forgo farm safety practices (i.e., helmets, rollbars, etc.). An integrated and holistic approach is essential to uncover the mental processes at play [82]. Psychologists, behavioral economists, and risk scholars could provide further insights into the underlying mental processes attributed to knowledge biases and risk-taking. Sociologists, anthropologists, and human geographers can provide insights into the circumstances under which these processes are likely to manifest. A consideration of the circumstances under which these mental processes occur is essential so that interventions can focus on addressing the root of the issue instead of the manifestations.

The third potential explanation is connected to competing demands for farmer resources and the availability of alternative options. The family farm literature has shown the many competing demands farm families face between the needs for financial and time resources of the household and the farm business [19,20,67,83]. Farmers in the early stages of their business, stages that have traditionally overlapped with the birth and early years of their children, tend to be in vulnerable positions due to high financial and mental stress [15,18,19,20,21,67]. In the context of our study, this might mean that the adoption of farm safety practices may not be economically feasible. Indeed, Hagel, Pahwa, Dosman and Pickett [57] and Elliot, Cammer, Pickett, Marlenga, Lawson, Dosman, Hagel, Koehncke and Trask [77] have pointed to the link between economic factors and the adoption of farm safety practices. Furthermore, while the supervision of children off the worksite is an important strategy to reduce children’s exposure to risk [2,44,84], farm parents face challenges towards accessing and paying for childcare, with a greater proportion of first-generation farmers reporting challenges than multi-generation farmers [7,14,85]. In our study, almost a quarter of the injuries were connected to playing or the home environment and considering previous research on pediatric injuries, [84] some of these injuries may be due to inadequate supervision of children by a working adult. Therefore, our findings, coupled with the existing literature, point to the need to consider ways in which underlying mechanisms associated with socio-economic inequalities and the policy environment (i.e., regulations, social and economic policies) have direct implications on farm parents’ ability to adopt farm safety practices [9,85,86]. Currently an understudied area in both the farm safety and broader farm family literature [7,14], there is a need to assess the role that childcare arrangements and childcare institutional supports may play in reducing the number of injuries among farm children. For example, are farm parents in countries with greater childcare support less likely to bring their children to the worksite? Additionally, considering the financial barriers to the adoption of safety practices [57,59,86], in what ways could farm equipment and infrastructure be doubled as safety structures for young children? While we are not aware of discussions around the staking of functions around farm safety, this idea has been suggested in the food safety literature to increase the adoption of practices by lowering the financial burden [86].

### 5.2. Differences between First- and Multi-Generation Respondents

The main differences between first- and multi-generation respondents was connected to demographic characteristics and parenting styles. Multi-generation respondents may have a greater level of resources due to their higher level of educational attainment and having fewer and older children. While Munden-Dixon, Tate, Cutts and Roche [54] did not find demographic differences between first- and multi-generation farmers in their sample, previous research has pointed to the greater level of challenges faced by first-generation farmers in the early years of operating their farm due to lower access to financial and social resources [51,52,55,87]. In terms of parenting, multi-generation respondents were more likely to have parenting styles with high involvement, namely authoritarian and permissive styles, which have previously been associated with a reduced risk of injury [35].

There are two main potential explanations to explain differences in the involvement levels of the two group of farm respondents. First, differences in childhood and socialization processes may explain some of the differences. Multi-generation respondents who grew up on the farm, where the home and workplace overlap, might be used to a high level of involvement from their parents, as they might have spent time in the workplace with a parent. First-generation respondents might have grown up in a household with parents working away from the home with less time to spend with the children during the workday. Additionally, Inwood, Clark and Bean [52] found that multi-generational farmers had a strong desire to pass the farm on to their children, while first-generation farmers were more focused on children choosing their own careers. This brings up questions about the ways in which differences in farm parents’ goals shape how they interact with their children, and the ways in which these differences may be reflected in their parenting style.

The second explanation for the differences in parenting style is connected to the differences in demographic and socio-economic characteristics. A closer look at the items that form the involvement construct (see details of the construct in Table 2) indicates that high involvement requires having time to spend with the children as well as having the knowledge to help them with schoolwork. While recent studies assessing farm parents’ parenting style did not control for the role played by socio-economic characteristics [35,36], Larson-Bright, Gerberich, Masten, Alexander, Gurney, Church, Ryan and Renier [75] pointed to the association between the mother’s education and off-farm work with the monitoring of children. Furthermore, the broader literature on parenting styles among the general population has extensively pointed to the association between socio-economic characteristics and parenting styles (see reviews of the literature by Hoff, et al. [88] and Roubinov and Boyce [89]). This is because educational, occupational, and financial factors affect access to resources, which in turn affect parental involvement. In our sample, multi-generational respondents had higher educational attainment, which is generally associated with higher socio-economic characteristics and higher levels of involvement. While these findings need to be reproduced on a larger sample, our findings bring up key questions about the extent to which interventions aimed at changing how farm parents parent are both feasible and may have negative unintended consequences. Several researchers have already pointed to the limited effectiveness of individual-level programming focused on knowledge deficits and behavior change to reduce exposure to risk on farms [9,23,59,60,61]. In particular, interventions that are incompatible with farmers’ economic realities have been found to be difficult to adopt for farmers with limited resources and to erode trust in farm safety experts’ advice. This points to the need to systematically consider how farmers’ lived realities and socio-economic characteristics may shape what they do with their children while they work. The rational is not to exonerate farmers from ensuring the safety of their children; it is to consider the full range of factors at play and the type of interventions that will be most effective in increasing the safety of children on farms.

### 5.3. Limitations

Our comparison of first- and multi-generation farmers led to the generation of rich insights for the farm safety literature as well as the generation of several avenues for future research. However, limitations connected to the sampling frame, sample size, and survey instrument mean that our study needs to be understood as exploratory with no claim of generalizability.

Starting with the limitations associated with our sampling frame, our use of private pesticide applicators and dairy producers lists from the PA and WI agencies of agriculture means we likely largely sampled farmers using conventional production practices. In previous studies that compared first- and multi-generational farmers, first-generation farmers were more likely to factor ecological factors in their farming goals and practices [52,53]. Furthermore, beginning farming programs targeted at new entrants tend to focus on sustainable growing practices [78,90]. Therefore, an interesting question to explore in future research is the intersectionality of farming background and production practices along a continuum of first- and multi-generation farmers. Inwood, Clark and Bean [52] provide a simple example of how multiple identities can be operationalized, even with a seemingly small sample, through the division of the sample across age group and farming background. The consideration of the continuum of first- and multi-generation farmers along growing practices is particularly important from the farm safety standpoint, as the exposure of children to risk likely varies due to differences in scale, commodities produced, and equipment and input used.

As discussed in our analytical strategy section, our sample size of 203 respondents (coupled with important missing variables, see next paragraph) did not support multivariate analysis. While adequate for exploratory research, our findings based on the bivariate analysis bring up two main points. First, if we had been able to control for respondents’ socio-economic backgrounds, the association between parenting style and farming background might have disappeared. However, we note the high chi-square value of the crosstab (Chi^2^ = 12.5). Second, the lack of statistical power could potentially explain the lack of statistically significant differences between the two groups around children injury, farm safety beliefs, knowledge, and practices.

The last limitations are connected to our survey instrument. First, we failed to collect information connected to the farm scale, growing practices, and household income. This limited our ability to develop an in-depth understanding of the differences between the two groups, compare our sample with previous studies, and assess the role of access to resources. Second, we collected data on confidence in farm safety knowledge vs. objective measures of farm safety knowledge. While asking about confidence in knowledge provides insights into beliefs, which in turn shapes practices, this brings up questions about the extent to which the disconnect between the adoption of practices and knowledge would still be present if we had used an objective measure.

## 6. Conclusions

Children growing up on farms are exposed to higher levels of risk compared to children from the general population [1,2,3,4]. In our study, about a third of farm parents reported that a child had suffered an injury at some point that required medical attention. Almost two-thirds of the injuries were connected to farming, while almost a quarter of the injuries were connected to playing or the home environment, which may in part be due to the inadequate supervision of children by a working adult. Farm safety scholars have long aimed to disentangle the factors associated with farm children’s risk exposure, but they have often done so from the venture point of farm parents who grew up on farms. The farm family scholars who compared farmers who grew up on farms with farmers who did not find key differences between these two groups connected to socialization into agriculture, access to resources, and farming knowledge and skills. In turn, these differences shape farm goals, farm structures, and the adoption of farm production practices [51,52,53,56]. Considering the importance of knowledge and access to resources in shaping exposure to risk [24,57], the goal of our article was to assess the extent to which differences on the basis of farming background may also affect the risk exposure of farm children. While exploratory in nature, our empirical comparison on the basis of farming background and the interpretation of the findings through the lens of two interdisciplinary and complementary bodies of literature, namely farm safety and farm family, led to the development of rich insights explaining the differences and similarities between the two groups of farm parent respondents.

Overall, our findings point to more similarities, including in the proportion of parents who reported a child injury, than differences between the first- and multi-generation farm parent respondents. We conclude our article with two key findings along with scholarly and practical implications. To be clear, these implications are grounded in our data and their limitations. Future research on larger samples and different sub-farm populations is needed to work towards generalizable patterns and middle-range theory [91]. First, as we highlighted in our Discussion section, the potential explanations for these similarities and differences point to a range of social, cultural, economic, and political factors across the multi-scalar agri-family system ranging from the micro level (i.e., deeply personal act of raising children, knowledge biases, differences in childhood and socialization processes) to the macro level (i.e., competing demands for farmer resources, limited availability of alternative options, and socio-economic conditions). This potential explanation is in contrast with the prominent individual-level explanations proposed in the farm safety literature. Second, the limited differences between first- and multi-generation respondents, despite likely differences in socialization processes, cultural values, and access to resources, might also indicate that while the farm population is heterogeneous (i.e., on the basis of differences in demographic, farm characteristics, and contextual differences), downward meso- and macro-level pressures placed on farmers means that their ability to adopt farm safety practices as well as their safety outcomes are heavily shaped by factors outside of their control [9,23,59]. Examples of downward meso- and macro-level pressures include agricultural commodity prices, high health insurance and health care costs, or the availability of amenities in rural areas, such as childcare.

Taken together, these findings illustrate and reaffirm the importance of working towards a holistic and systemic understanding of the factors that shape farm parents’ decisions and their children’s exposure to farm risk. As illustrated in our introduction, farm safety scholars have long embraced micro-level analyses of farm families. Yet, we argue that these micro-level analyses are most productive when they integrate farm families’ lived realities, and when these micro-level analyses are connected with the broader factors that shape farm parents’ characteristics, their lived realities, and the farm safety choices they make. This is a common approach in social science disciplines such as sociology, anthropology, and human geography. Janssen and Nonnenmann [59], Shortall, McKee and Sutherland [9], and Thu [23] provide examples of this approach applied to farm safety. While beyond the scope of this paper, we note that the publication of these articles in social science journals over farm safety journals means the diffusion of their approach and findings to the broader farm safety field might have been limited. This raises questions about how disciplinary silos can be broken down to increase the diffusion and integration of knowledge.

Lastly, our findings illustrate and reaffirm the importance of expanding our toolkit of interventions to address meso- and macro-level factors. Lee, Bendixsen, Liebman and Gallagher [58] have previously made this argument, yet this call has seldom been answered [61,92]. The enforcement of existing, and the addition of, strong safety and labor regulations is perhaps what farm safety experts’ most often recommend. While stronger regulations are an important avenue towards decreasing children’s exposure to risk, there is a need to assess the potential unintended consequences of such an approach, considering the power imbalance in the agricultural sector and the extent to which farmers’ agency may be limited by factors outside of their control [23,59,93,94]. Another important area for intervention would be to address social and economic conditions in agriculture at the farm operation and/or farm household level. This could be achieved through interventions aimed at supporting the economic viability of farm operations by addressing challenges associated with inadequate commodity prices and concentration and consolidation in the agricultural sector. This type of intervention may be novel for the farm safety field. However, there is evidence that agricultural policies shape farmers’ health outcomes [23,95,96]. Instead of starting afresh, there are extensive opportunities for farm safety scholars to connect with disciplines that have long traditions of studying agricultural policies such as agricultural economics and rural sociology to understand the current structure of agricultural policies, the ways in which they could be adjusted, and agricultural regulations and supports that could best support farm safety. Social and economic conditions in the agricultural sector could also be addressed through interventions focused on increasing the social and economic well-being of farm families [97,98]. This is particularly important in the US, considering farm parents’ high level of challenges with affordability and accessibility of childcare before and during COVID-19 [7,14,85]. As social and economic conditions of farm families have received much less attention in the literature compared to the economic conditions of farm operations, Becot and Inwood [97] recently outlined a research agenda to integrate household needs and social policy into the international family farm research agenda. Ultimately, interventions to address challenges at the meso and macro levels may not only reduce farm risk; they could also more broadly support the sustainability and resilience of farm labor systems.

## Figures and Tables

**Table 1:** Survey measures.

Survey Measures	Measurement	Recoding
Demographics and Farm Characteristics		
Farming background	1. First-generation; 2. Multi-generation	Variable created using question asking which generation respondent was from.
Gender	1. Male, 2. Female	No recoding.
Age	Continuous—range of responses: 23–71	No recoding.
Educational attainment	1. Less than high school; 2. High school degree; 3. Two-year college degree or more	Collapsed from four categories.
Number of children	Continuous—range of responses: 1–17	New variable based on question asking information for each children.
Have children under the age of 7	1. Yes; 0. No	New variable based on question asking information for each children.
Have off-farm job	1. Yes; 0. No	Collapsed from three categories (full-time, part-time, no off-farm job).
Beginning farmer status	1. Yes; 0. No	Variable created using number of years farming with ≤10 years as beginning farmer threshold.
Weekly hours worked on the farm	Continuous—range of responses: 0–115	No recoding.
Primary commodity produced (Field crops, dairy/beef, Vegetable/fruit/ nursery, other)	1. Yes; 0. No.	Dairy or beef variable was created using two separate variables, other was created using four separate variables (swine, sheep, poultry, other). No recoding for field crops and vegetable/fruit/ nursery.
Children safety measures		
At least one child had suffered injury that required medical attention	1. Yes; 0. No	No recoding.
Compared to other occupations, agriculture is:	1. More safe or equally as safe; 2. Less safe.	Collapasped from 3 to 2 categories.
Parents’ confidence in ability to supervise farm work and confidence in safety knowledge	Continuous—range of responses: 1–4	New variables created by summarizing five survey items. See Table 2 for item details.
Children participate in farm work	1. Yes; 0. No	New variable based on question asking information for each children.
Use of invisible and physical boundary play area	1. Current use or intention to use in the future; 2. No current use and no intention to use in the future	Collapsed from 5 to 2 categories.
Parenting styles	1. Authoritative (high on involvement and high on control); 2. Uninvolved (low on involvement and low on control); 3. Authoritarian (low on involvement and high on control); 4. Permissive (high on involvement and low on control)	New variable created by using the mean threshold of parental involvment and control dimensions. See text and Table 2 for list of items and recoding approach.

**Table 2:** Confidence in farm safety knowledge and parenting style survey constructs.

Constructs	Construct Items
Confidence in farm safety knowledge	
Parents’ confidence in ability to supervise farm work (α = 0.92)	Feel confident assigning physically appropriate agricultural tasks/chores to my child based on their physical capabilities. Feel confident assigning mentally appropriate agricultural tasks/chores to my child based on their mental capabilities. Feel confident establishing and enforcing rules regarding safe agricultural work practices to my child. Feel confident providing adequate supervision while my child performs agricultural tasks/chores. Feel confident training my child to safely perform the agricultural tasks/chores I assign.
Parents’ confidence in safety knowledge(α = 0.87)	Feel confident assigning the correct personal protective equipment (e.g., gloves, goggles, hearing protection, respirators) to my child for specific agricultural tasks/chores. Feel confident enforcing the use of personal protective equipment (e.g., gloves, goggles, hearing protection, respirators) by my child while performing specific agricultural tasks/chores. Feel confident identifying the safety and health hazards of agricultural tasks/chores. Feel confident removing the safety and health hazards of agricultural tasks/chores prior to assigning the task to my child. Know where I can find quality materials to assist in assigning, supervising, and training my child on agricultural tasks/chores.
Parenting styles dimensions	
Involvement	When someone within our family comes home or leaves home, he/she lets other family members know. I encourage my child to try harder when he/she receives a poor grade in school. I help my child with an assignment that he/she does not understand. My child can count on me when he/she has some kind of problem. I find it very easy to talk openly with my child. I spend time just talking with my child. When my child receives a good grade in school I show him/her my approval. We do things for fun together regularly as a family. When my child gets a poor grade I suggest to help him/her.
Control	I really know what my child does in his/her free time. I try to know where my child is in the afternoon after school. I really know where my child goes at night. I really know where my child is in the afternoon after school. I try to know where my child goes at night. I try to know what my child does in his/her free time.

Notes: Survey items adapted from [22,70,71].

**Table 3:** Descriptive statistics.

	All (*n* = 203)	First-Gen (*n* = 64)	Multi-Gen (*n* = 139)	F-Statistic/Chi2; *p* Value
Farming background (%)		32.0	68.0	N/A
Gender (%)				3.7; *p* = 0.05
Male	77.1	68.8	81.0	
Female	22.9	31.2	19.0	
Age (mean and standard deviation in years)	44.2 (10.8)	43.9 (10.3)	44.3 (11.2)	0.03; *p* = 0.86
Educational attainment (%)				35.1; *p* = 0.00
Less than High school	46.7	77.4	32.8	
High school degree	28.1	8.1	37.2	
Two-year college degree or more	25.1	14.5	29.9	
Children on the farm				
Number of children (mean and standard deviation in years)	3.1 (2.2)	3.7 (2.5)	2.7 (1.9)	9.7; *p* = 0.00
Have children under the age of seven (%)	52.3	63.3	47.4	4.2; *p* = 0.04
Have off-farm job (%)	28.7	20.3	32.6	3.2; *p* = 0.07
Beginning farmer status (%)	20.3	23.8	18.7	0.7; *p* = 0.40
Weekly hours worked on the farm (mean and standard deviation in hours)	59.9 (28.6)	59.7 (27.3)	59.4 (29.5)	0.0; *p* = 0.96
Primary commodity produced (%)				7.9; *p* = 0.05
Field crops	18.3	7.8	23.2	
Dairy or beef	65.8	71.9	63.0	
Vegetable, fruit, or nursery	7.4	10.9	5.8	
Other	8.4	9.4	8.0	

Note. Race and ethnicity not reported in table as 99.5% of the sample was white non-Hispanic.

**Table 4:** Comparison of children safety measures across first- and multi-generational farmers.

	All (*n* = 203)	First-Gen (*n* = 64)	Multi-Gen (*n* = 139)	F-Statistic/Chi2; *p* Value
Previous experience with farm injury				
At least one child had suffered injury that required medical attention (%)	34.4	38.3	32.6	0.6; *p* = 0.44
Farm safety beliefs				
Compared to other occupations, agriculture is (%):				0.2; *p* = 0.67
More safe or equally as safe	67.2	65.0	68.2	
Less safe	32.8	35.0	31.9	
Confidence in farm safety knowledge (mean and standard deviation—scale of 1 to 4)				
Parents’ confidence in ability to supervise farm work	3.5 (0.5)	3.4 (0.4)	3.5 (0.5)	2.96; *p* = 0.09
Parents’ confidence in safety knowledge	3.3 (0.5)	3.2 (0.5)	3.4 (0.5)	2.54; *p* = 0.11
Children participate in farm work (%)	74.9	79.7	72.7	1.1; *p* = 0.28
Use of invisible boundary play area (%)				0.1; *p* = 0.74
Current use or intention to use in the future	97.3	97.9	97.0	
No and no intention to use in the future	2.7	2.1	3.0	
Use of physical boundary play area (%)				0.4; *p* = 0.54
Current use or intention to use in the future	36.1	39.6	34.4	
No use and no intention to use in the future	63.9	60.4	65.6	
Children participate in farm work (%)	74.9	79.7	72.7	1.1; *p* = 0.28
Parenting styles (%)				12.5; *p* = 0.01
Authoritative	41.7	23.5	50.5	
Uninvolved	34.0	49.0	26.7	
Authoritarian	14.7	19.6	12.4	
Permissive	9.6	7.8	10.5	

## Data Availability

The data presented in this study are available on request from the corresponding author. The data are not publicly available due to open-ended questions that could identify respondents and to authors’ institution requiring a data sharing agreement before research data may be shared.
